# Terminal Platelet Production is Regulated by Von Willebrand Factor

**DOI:** 10.1371/journal.pone.0063810

**Published:** 2013-05-30

**Authors:** Sonia Poirault-Chassac, Kim Anh Nguyen, Audrey Pietrzyk, Caterina Casari, Agnes Veyradier, Cecile V. Denis, Dominique Baruch

**Affiliations:** 1 INSERM UMR 765, Paris, France; 2 Université Paris Descartes, Sorbonne Paris Cité, Paris, France; 3 INSERM UMR 770, Le Kremlin-Bicetre, France; 4 Univ Sud Paris, Le Kremlin-Bicetre, France; 5 Antoine Béclère Hospital and National Reference Center for von Willebrand disease, Clamart, France; Julius-Maximilians-Universität Würzburg, Germany

## Abstract

It is established that proplatelets are formed from mature megakaryocytes (MK) as intermediates before platelet production. Recently, the presence of proplatelets was described in blood incubated in static conditions. We have previously demonstrated that platelet and proplatelet formation is upregulated by MK exposure to high shear rates (1800 s^−1^) on immobilized von Willebrand factor (VWF). The purpose of the present study was to investigate whether VWF is involved in the regulation of terminal platelet production in blood. To this end, *Vwf ^−/−^* mice, a model of severe von Willebrand disease, were used to create a situation in which blood cells circulate in a vascular tree that is completely devoid of VWF. Murine platelets were isolated from *Vwf ^−/−^* and *Vwf^ +/+^* blood, exposed to VWF at 1800 s^−1^ in a microfluidic platform, and examined by means of videomicroscopy, as well as fluorescence and activation studies. Proplatelets became visible within 5 minutes, representing 38% of all platelets after 12 minutes and 46% after 28 min. The proportion of proplatelets was 1.8-fold higher in blood from *Vwf^−/−^* mice than from *Vwf^+/+^* mice, suggesting a role of VWF *in vivo*. Fragmentation of these proplatelets into smaller discoid platelets was also observed in real-time. Platelets remained fully activatable by thrombin. Compensation of plasmatic VWF following hydrodynamic gene transfer in *Vwf^−/−^* mice reduced the percentage of proplatelets to wild-type levels. A thrombocytopenic mouse model was studied in the flow system, 7 days after a single 5-FU injection. Compared to untreated mouse blood, a 2-fold increase in the percentage of proplatelets was detected following exposure to 1800 s^−1^ on VWF of samples from mice treated with 5-FU. In conclusion, VWF and shear stress together appear to upregulate proplatelet reorganization and platelet formation. This suggests a new function for VWF *in vivo* as regulator of bloodstream thrombopoiesis.

## Introduction

Von Willebrand factor (VWF) is a multimeric protein synthesized by endothelial cells and stored in Weibel Palade bodies, from which it is released into the circulation [1]. VWF is produced as ultra-large multimers associated with endothelial cells on the luminal side of blood vessels and cleaved from the cell membrane by the metalloprotease ADAMTS-13, resulting in circulating VWF multimers of different sizes. VWF synthesized by endothelial cells is also secreted constitutively into the subendothelium on the basal side of endothelial cells and associates with extracellular matrix components [2]. VWF is also synthesized by megakaryocytes (MKs) and stored in platelet α-granules. The main function of VWF is to form an adhesive surface that initiates platelet arrest at sites of vascular injury, followed by platelet activation and thrombus formation [3], [4]. VWF binds via its A1 domain to the platelet receptor glycoprotein (GP) Ib-V-IX, at the N-terminal end of the GPIbα subunit [5]. A striking feature of GPIb-mediated platelet rolling on VWF is that it occurs at high shear rates: VWF seems to be unique among adhesive proteins, in that it undergoes a conformational change when exposed to high shear rates [6]. This structural reorganization allows high shear rates to promote the reversible interaction between VWF and GPIb, leading to platelet translocation that is sustained as long as the αIIbβ3 integrin remains in a non-activated state. There is clear evidence that only a fraction of GPIb-binding sites of freshly secreted VWF on endothelial cells are occupied, leaving ample possibilities for rapid regulatory mechanisms to increase the part of available binding sites for VWF [7]. One such mechanism is an increase in hemodynamic shear forces.

Hematopoietic stem cells proliferate in bone marrow and differentiate into several lineages. Megakaryopoiesis is a complex mechanism that gives rise to mature MKs characterized by their large size, polyploidy, and an extended cytoskeleton known as the demarcation membrane system. Thrombopoiesis is the final stage of MK development and results in platelet formation [8]. Contrary to the proliferation and maturation of MK precursors, thrombopoiesis does not take place in bone marrow but rather requires the passage of large MK fragments into sinusoid vessels present in bone marrow. While most studies of platelet production have been performed in static conditions, the importance of blood flow is increasingly recognized. Clear evidence of the role of blood flow in MK fragmentation has been obtained by means of intra-vital videomicroscopy of mouse bone marrow, showing transendothelial passage of large MK fragments and their breakdown into smaller fragments along sinusoid vessels [9]. These MK fragments are carried by the bloodstream into the venous circulation, where their fragmentation continues to yield proplatelets, suggesting that shear forces exerted on vessel walls may be involved in platelet production outside the bone marrow environment. Another site of thrombopoiesis is the lung capillary circulation, where MK nuclei were first observed several decades ago [10]. Intravenously infused MKs have been shown to localize specifically in the lung and to shed platelets [11]. It is interesting to note that lung capillary is the first vascular bed exposed to high shear rates that MK fragments encounter on leaving the bone marrow circulation [12].

We have recently identified a new mechanism whereby human MK exposure to high shear rates in an *ex vivo* flow model accelerates proplatelet formation and platelet release [13]. VWF was required in this system for GPIb-mediated MK adhesion and translocation. MK then underwent microtubular rearrangements to form proplatelets, breaking into several elements still linked to one another by tubulin shafts; finally, the release of single, isolated platelets was visualized for the first time in dynamic conditions [13]. This sequence of events in the presence of shear forces, in which proplatelet reorganization leads to platelet generation, was later confirmed by Thon et al., who reported cytoskeletal remodeling of fetal liver MK fragments into proplatelets and platelets [14], [15]. While it is commonly admitted that circulating platelets represent terminally fragmented products that are devoid of a nucleus, a recent publication provides insights into *ex vivo* processing of peripheral blood platelets [16]. Human washed platelets that were maintained *ex vivo* for several hours were able to change their morphology so as to resemble dumbbell-shaped proplatelets. These modified platelets were thought to be involved in platelet duplication and in the formation of so-called platelet progeny [16]. Similar findings were made when platelets were kept in whole blood in the absence of hemodynamic flow [16].

The aim of this study was to further dissect the terminal stages of platelet production, focusing on the role of shear stress and VWF in the transition between proplatelets and discoid platelets. We therefore examined whether perfusion, on VWF and at a high shear rate, of platelets isolated *ex vivo* from human and mouse blood samples permitted real-time observation of proplatelet fragmentation into single platelets, as we have previously observed using cultured MK [13]. We used platelets from *Vwf ^−/−^* mice, an established model of severe von Willebrand disease [17]. Not only do platelets from these mice lack VWF in their α-granules, but their blood cells also circulate in a vascular tree that is completely devoid of VWF.

## Materials and Methods

### Ethics Statement

The patient with von Willebrand disease gave written informed consent for blood collection, in keeping with the ethical agreement related to the National Reference Center for von Willebrand Disease (acronym CRMW: Centre national de Référence de la Maladie de Willebrand) coordinated by Professor Agnès Veyradier and approved by the French Ministry of Health in 2006. CRMW organizes a national platform dedicated to the analysis of blood samples from patients with von Willebrand disease. Authorization for data analysis was obtained from the computer watchdog authority (CNIL) by Service d’Hématologie Biologique of Antoine Béclère hospital on behalf of CRMW. Animal studies received ethical approval from Comité d’Ethique pour l’Experimentation Animale Paris Descartes (#CEEA34.CBV.013.11). Animal suffering was estimated to be weak.

### Patient

Blood was drawn on acid citrate dextrose solution (ACD) from a patient with inherited von Willebrand disease type 3 (phenotype: undetectable plasma VWF levels, genotype: homozygosity for a small insertion within exon 42 of *VWF*, creating a stop codon within the B3 domain of VWF) at Antoine Béclère Hospital (Clamart, France). The patient did not have alloantibodies and had not received any VWF concentrates during the previous 12 weeks. Control blood was drawn on ACD from a healthy volunteer.

### Mice

*Vwf ^−/−^* and *Vwf* ^+/+^ mice [17] on a pure C57Bl/6J background were used between 8 and 12 weeks of age. For shear experiments, blood was sampled by cardiac puncture and was used immediately. Hydrodynamic gene transfer of murine VWF cDNA to *Vwf ^−/−^* mice was performed as previously described, resulting in increased plasma VWF:Ag levels for at least one week [18]. Platelets were exposed to shear forces 4 days after hydrodynamic injection. Thrombocytopenia was induced by a single intraperitoneal injection of 150 mg/kg 5-fluorouracil (5-FU), and platelet counts and median volumes were recorded daily with a MS9-3 device (Melet Schloesing Laboratories, Cergy-Pontoise, France) for the following 11 days. For shear experiments, thrombocytopenic mouse blood was taken 7 days after 5-FU injection.

### Platelet Isolation

Mouse blood was collected on ACD-C (124 mmol/L Na_3_ citrate, 130 mmol/L citric acid pH 6.5, 110 mmol/L dextrose) and platelets were isolated by sequential centrifugation. Briefly, platelet-rich plasma was obtained by centrifugation for 7 min at 160 *g*, red cells were removed by a short centrifugation step (2 min, 110 *g*) and platelets were washed in buffer (36 mmol/L citric acid, 103 mmol/L NaCl, 5 mmol/L KCl, pH 6.5, containing 5 mmol/L glucose, 2 mmol/L CaCl_2_, 1 mmol/L MgCl_2_). Following centrifugation (10 min, 750 *g*), platelets were resuspended at a density of 270 G/L in modified Hepes-Tyrode buffer (137 mmol/L NaCl, 2 mmol/L KCl, 0.3 mmol/L NaH_2_PO_4_, 12 mmol/L NaHCO_3_, 1 mmol/L MgCl_2,_ pH 7.3). Prostaglandin E1 (1 µmol/L) and apyrase (100 mU/mL), both from Sigma-Aldrich (Lyon, France), were added at all but the first and last steps. Platelets were counted with an MS9-3 counter. Human blood was collected on ACD from volunteers, and washed platelets were obtained by standard procedures within 2 to 3 hours. Platelet counts were adjusted to 270 G/L in an electronic particle counter (Model Z1, Coulter Electronics, Margency, France) [19].

### Flow Assay

Platelets were perfused via an electropneumatic pump through a Bioflux200 microfluidic platform (Fluxion Bioscience, San Francisco, CA) [20] consisting of VWF-coated glass microchannels, at a shear rate of 1800 s^−1^. In some experiments, washed mouse platelets were perfused on murine recombinant VWF (30 µg/mL), produced as a baby hamster kidney (BHK) cell supernatant. Prior to flow experiments, murine VWF was immobilized overnight on a surface precoated for 2 hours with a polyclonal anti-human VWF antibody (10 µg/mL, Dako, Trappes, France). In some experiments, purified human VWF (40 µg/mL), fibronectin (20 µg/mL) or fibrinogen (20 µg/mL) was directly adsorbed to the coverslip, as previously reported [13]. Real-time adhesion was observed in the same microscopic field at 40X magnification over a 35-min period, starting 4 minutes after the onset of perfusion. The stage was kept at the same position and digital images were recorded (0.25 image/second) with a CCD camera (Sony and Q-click Imaging) and Replay software (Microvision Intruments, Evry, France). Histolab software (Microvision Intruments) was used to monitor changes in platelet morphology at 30-second intervals. Digital images were recorded for 30 minutes after the start of the perfusion, in order to obtain quantitative data, using Videomet software (Microvision Intruments). Morphometric parameters, recorded by considering the platelet as an ellipse or manually drawing its contours, comprised the surface area, perimeter, Feret X (largest dimension) and Feret Y (shortest dimension). In some experiments, platelets in contact with the VWF surface inside microchannels were visualized in real time after 40 min of flow. To this end, live mouse platelets were stained with 250 nmol/L paclitaxel coupled to Oregon green (Tubulin-Tracker green reagent, Molecular Probes) to allow binding to polymerized tubulin. In other settings, human platelets were activated with 1 U/mL bovine thrombin (Sigma-Aldrich) in the presence of 2 mmol/L CaCl_2_ for 10 minutes, then fixed with paraformaldehyde (PFA) and incubated with an Alexa Fluor 488-conjugated mouse anti-human CD62P antibody (clone AK4, Biolegend). After washing with phosphate buffer saline (Invitrogen, Saint-Aubin, France), fluorescence was analyzed at 494 nm and 522 nm (absorption and emission, respectively), using a high-resolution bioimaging platform (EMCCD MGi Plus Qimaging Rolera camera, Roper Scientific, Evry, France). Prior to or following shear exposure, an aliquot of human platelets was layered on polylysine-coated coverslips. Platelet actin and tubulin were analyzed by means of confocal immunofluorescence imaging in static conditions as previously reported [13].

### Flow Cytometry

Following shear exposure, an aliquot of mouse platelets was collected at the exit of the flow chamber, then activated with 1 U/mL bovine thrombin (Sigma-Aldrich) in the presence of 2 mmol/L CaCl_2_ for 10 minutes and incubated with FITC-conjugated rat anti-mouse CD62P antibody or an isotype control with a similar conjugate (Emfret Analytics, Würzburg, Germany). Platelets kept in static conditions were compared after similar activation by thrombin. Alternatively, fibrinogen coupled to Alexa 488 (InvitroGen) was used at 2 µg/mL. Platelets were fixed with PFA, then washed and analyzed in a Facscalibur flow cytometer (Becton-Dickinson).

### Statistical Analysis

Results are expressed as means ± SEM and were compared by using Student’s *t* test for unpaired samples. P values <0.05 were considered to denote statistical significance.

## Results

### Ex vivo Perfusion on VWF at High Shear Rates Reveals the Presence of Reversibly Adherent Proplatelets and Platelets

**Figure 1:**
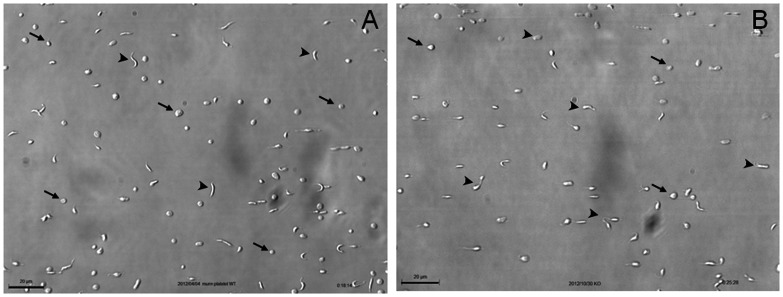
Characterization of proplatelets. Platelets were isolated from mouse blood and perfused on murine VWF at 1800 s^−1^. Reversibly adherent platelets appeared as discoid (arrows) or as proplatelets displaying an extended, dumbbell-shaped or multibodied morphology (arrowheads). All these different morphological states were observed with platelets from *Vwf^ +/+^* (Panel A) and *Vwf ^−/−^* (Panel B) mouse blood. Bar = 20 µm.

To study the role of VWF in the terminal stages of platelet production, we used a murine model of complete VWF deficiency in all vascular and cellular compartments [17]. Washed platelets isolated from blood of wild-type (*Vwf^ +/+^*, Figure 1A) and *Vwf ^−/−^* mice (Figure 1B) were perfused *ex vivo* on a murine-VWF-coated surface at a shear rate of 1800 s^−1^, and platelet adhesion was recorded continuously for 30 minutes. Importantly, platelets isolated from mice of both genotypes presented short reversible contacts, indicating platelet translocation on VWF. As seen on still frames (Figure 1) and video recordings (Video S1 and S2), these reversibly adherent platelets exhibited a variety of morphological features. Beside small discoid platelets, we observed proplatelets with elongated shapes (dumbbell, “figure 8″, or multibodied elements with one or two extensions). These proplatelets were reminiscent of those generated by MK fragmentation reported in previous studies from our team [13] and others [14], [15]. Interestingly, discoid platelets and proplatelets kept moving along the surface, indicating that they were not activated (Video S1 and S2). The use of a microfluidic system at a high shear rate not only allowed proplatelets and platelets to be captured on VWF by keeping them in a reversibly adherent condition, but also enabled morphological transitions of proplatelets to be observed (Video S2), as well as the direct release of a small discoid platelet from a translocating proplatelet shown on a sequence indicated by the simultaneous presence of an arrow and an arrowhead (Video S1).These results suggested that blood of both *Vwf^ +/+^* and *Vwf ^−/−^* mice contained a mixture of proplatelets and terminally processed discoid platelets.

In some experiments, platelets were perfused on VWF at lower shear rates (600 and 1200 s^−1^) and with a longer perfusion time, in order to ensure similar perfusate volumes. Discoid platelets and proplatelets were observed moving along the surface, but some activation took place, as shown by the increased number of spread platelets at 600 and 1200 s^−1^ (data not shown). Thus, a shear rate of 1800 s^−1^ was used for subsequent VWF adhesion experiments. In other experiments with a shear rate of 1800 s^−1^, fibronectin or fibrinogen was coated on the surface but no platelet translocation was observed on these substrates at such high shear rate (data not shown).

#### Different microtubule organizations of proplatelets and platelets depend on the presence of VWF in vivo and are modified by exposure to a high shear rate ex vivo

**Figure 2:**
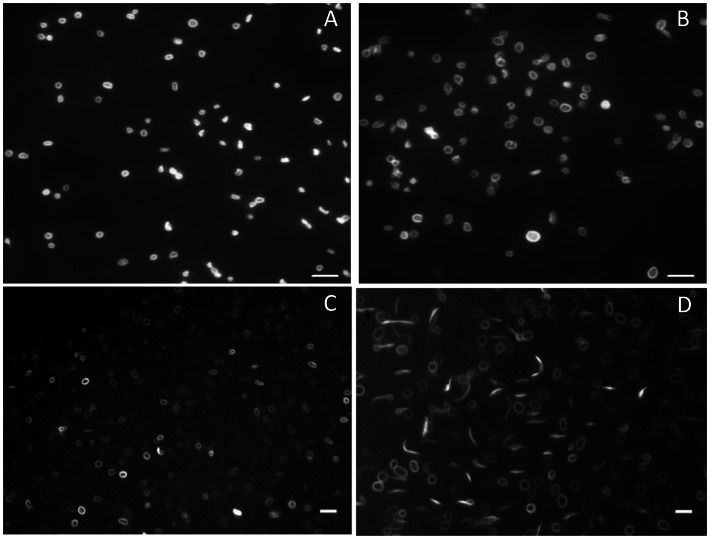
Tubulin staining of reversibly VWF-adherent platelets visualized in real time. Platelets were isolated from *Vwf^ +/+^* (Panels A, C) and *Vwf ^−/−^* (Panels B, D) mouse blood and tubulin staining was studied prior to (Panels A, B) or during perfusion on VWF at 1800 s^−1^ for 40 min (Panels C, D). Then tubulin staining was monitored in the flow channels by incubation of platelets with tubulin tracker (Panels C, D). *Vwf^ +/+^* blood contained a majority of discoid platelets (Panels A, C), and no proplatelet was visible in the static sample (Panel A). In contrast, *Vwf ^−/−^* blood (Panel B, D) contained a mixture of dumbbell-shaped proplatelets and platelets with a circular ring of tubulin. Proplatelets were more abundant in the shear sample (Panel D) than in the static sample, with one “figure 8” proplatelet clearly visible (Panel B). Notice that *Vwf ^−/−^* platelets were larger than *Vwf^ +/+^* platelets. Images were recorded using a Qimaging Rolera camera. Bar = 10 µm.

We have previously reported that tubulin polymerization is necessary for proplatelets to reorganize and break up into platelets [13]. Microtubule organization of washed platelets was studied prior to, as well as during, shear exposure (Figure 2). A variety of microtubule organizations was observed: in pre-shear samples, *Vwf^ +/+^* platelets were consistently seen as discoid platelets with a double tubulin ring and no proplatelet was visible in the static sample (Figure 2A). In contrast, *Vwf ^−/−^* blood contained a mixture of proplatelets and platelets (Figure 2B). In order to characterize the cytoskeletal organization of platelets exposed to a high shear rate on VWF, we used a direct staining procedure *in situ* in the flow channel. At the end of the perfusion period on VWF, live proplatelets and platelets were incubated with paclitaxel (in order to stabilize microtubules) coupled to an intra-vital probe, and flow was continued in order to visualize polymerized tubulin. In *Vwf^ +/+^* samples, a few proplatelets were seen together with discoid platelets (Figure 2C). However, in adherent *Vwf ^−/−^* samples, proplatelets displaying microtubules organized in a variety of shapes (dumbbell, bean, comma, or multibodied, i.e. composed of an elongated thread linking one or two bulges in the center), were visible, as well as discoid platelets that were larger than those seen in *Vwf^ +/+^* samples (Figure 2D). This suggested that 1) in static conditions, proplatelets in *Vwf^+/+^* samples were rare events, 2) *Vwf ^−/−^* contained more proplatelets than *Vwf^ +/+^* samples prior to the flow, 3) exposure to high shear stress allowed visualizing more proplatelets than in static conditions, and 4) that *Vwf ^−/−^* compared to *Vwf^ +/+^* contained more proplatelets than *Vwf^ +/+^* in shear conditions.

**Figure 3:**
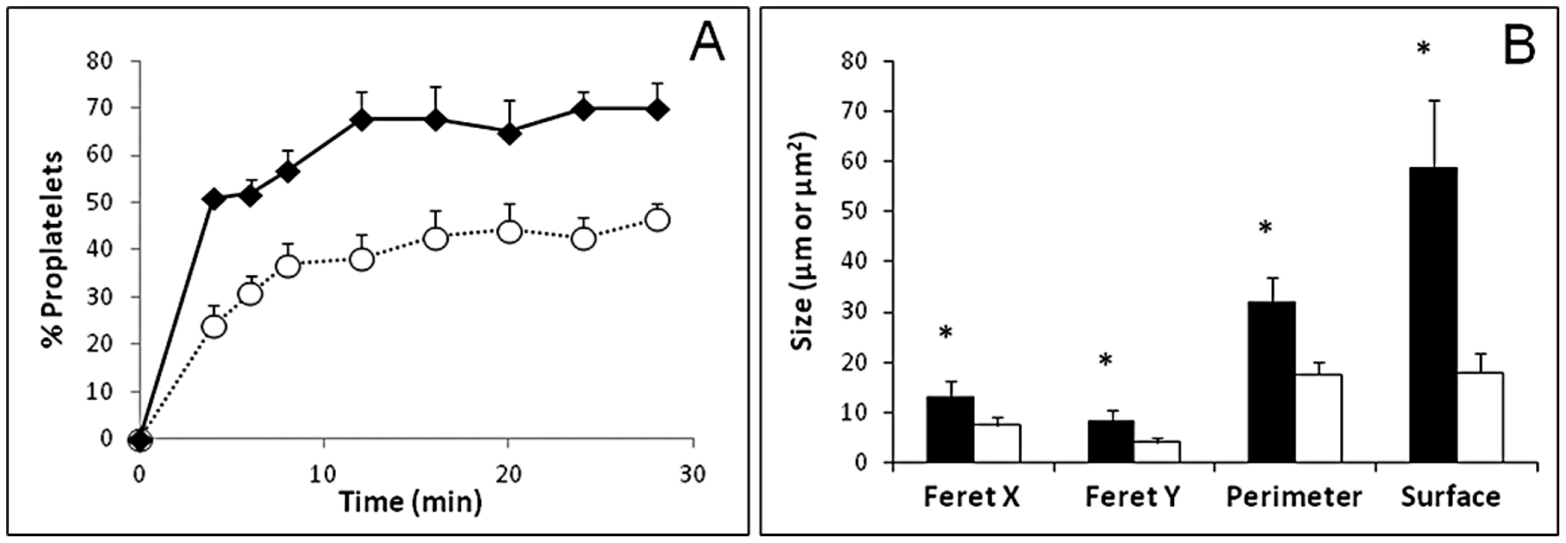
Quantification of proplatelets in *Vwf^ +/+^* and *Vwf ^−/−^* mouse blood. Platelets isolated from *Vwf^ +/+^* and *Vwf ^−/−^* mouse blood were perfused on murine VWF at 1800 s^−1^. Panel A: once the density of platelets flowing on the surface became homogenous (4 min after the beginning of flow), translocating proplatelets and discoid platelets were counted by real-time videomicroscopy. The % of proplatelets was calculated as described in the Results section for *Vwf ^−/−^* platelets (closed diamonds) and *Vwf^ +/+^* platelets (open circles). Means and SEM of 4 experiments are shown. Panel B: Quantitative morphometry of Feret X, Feret Y, perimeter and surface area of adherent discoid platelets and proplatelets from *Vwf ^−/−^* (black bars) vs *Vwf^ +/+^* mouse blood (white bars), as described in Material and Methods. *P<0.0001.

VWF thus appears to be required for the completion of proplatelet fragmentation into platelets. To obtain quantitative data, we classified translocating platelets as either discoid platelets (class 1) or proplatelets (class 2), based on the shapes described above. The percentage of proplatelets was calculated as 100 * (number of class 2)/[(number of class 1)+(number of class 2)]. Spread platelets and small aggregates were also counted, as an indicator of activation. Fewer than 5% of total adherent cells were activated platelets in all experiments. The percentage of proplatelets increased with time until a plateau was reached, corresponding to the time necessary for the bulk of platelets to reach equilibrium inside the flow channel and to be captured on the surface. A significantly higher percentage of proplatelets was found at all time points in *Vwf ^−/−^* samples than *Vwf^+/+^* samples (p<0.05): indeed, from 4 min to 28 min, the % of newly formed *Vwf ^−/−^* platelets increased from 50.88±0.88 to 69.97±5.26, while the % of newly formed *Vwf^ +/+^* platelets increased from 23.92±4.24 to 46.46±3.37 (Figure 3A). Morphometric study of reversibly adherent platelets indicated that *Vwf ^−/−^* platelets were significantly larger than *Vwf^ +/+^* platelets (p<0.0001), whether based on the perimeter, surface area, Feret X or Feret Y values (Figure 3B).

### Compensation of VWF Plasma Concentrations in Vwf ^−/−^ Mice in vivo Modifies the Proplatelet to Platelet Transition in Blood

**Figure 4:**
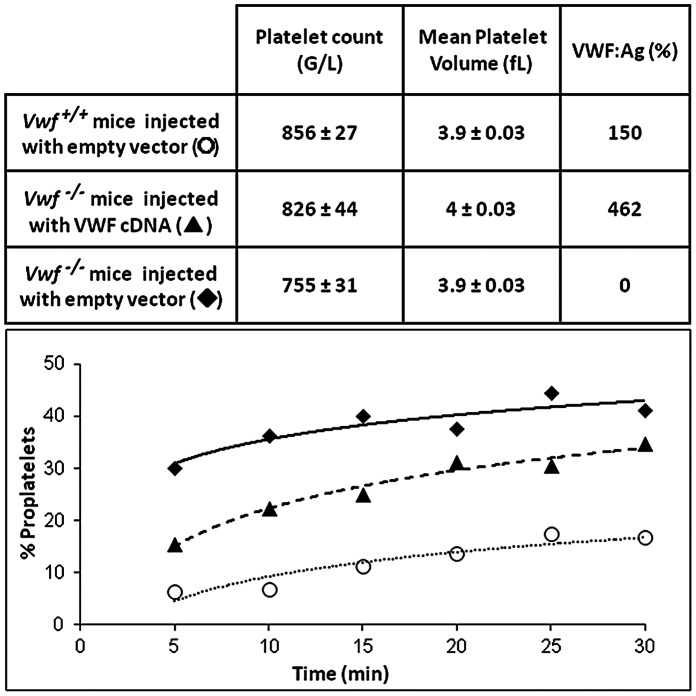
Hydrodynamic injection of VWF cDNA in a murine model of von Willebrand disease. *In vivo* injection of VWF cDNA in VWF-deficient mice, and of a control plasmid (pLIVE) in *Vwf ^−/−^* and *Vwf^ +/+^* mice, was performed 4 days before *ex vivo* adhesion experiments. Quantification of proplatelets was performed as described in the legend of Figure 3. The % of proplatelets is shown for platelets from *Vwf ^−/−^* mice injected with the empty vector (closed diamonds), *Vwf ^−/−^* mice injected with VWF cDNA (closed triangles) and *Vwf^ +/+^* mice injected with the empty vector (open circles). The upper table displays platelet counts, mean platelet volumes and VWF:Ag % in blood samples from the corresponding mice, 4 days after plasmid injection.

Hydrodynamic injections of VWF cDNA in *Vwf ^−/−^* mice have been reported to compensate for the lack of plasmatic VWF but not to replenish its vascular stores [18], [21]. We used this approach to investigate how restored VWF plasma levels affected proplatelet and platelet formation in *Vwf ^−/−^* mice. Mean VWF:Ag levels in these mice reached 462% following injection. To evaluate the effect of hydrodynamic injection itself, *Vwf ^−/−^* and *Vwf ^+/+^* mice were injected with the empty vector. A non specific increase in VWF:Ag was observed in *Vwf ^+/+^* mice (up to 150%). Interestingly, the % of proplatelets in *Vwf ^−/−^* mice injected with VWF cDNA was lower than in control *Vwf ^−/−^* mice injected with the empty vector, but it remained higher than that observed in *Vwf^ +/+^* mice injected with the empty vector (Figure 4). Together, these results suggested that circulating blood (pro)platelets may lack a part of the thrombopoiesis process in *Vwf ^−/−^* mice compared to *Vwf^+/+^* mice, and that this defect is partially compensated for by the presence of VWF in plasma and adsorbed on the endothelial surface.

#### Proplatelets and platelets translocating on VWF at a high shear rate display fully thrombin- activatable status

**Figure 5:**
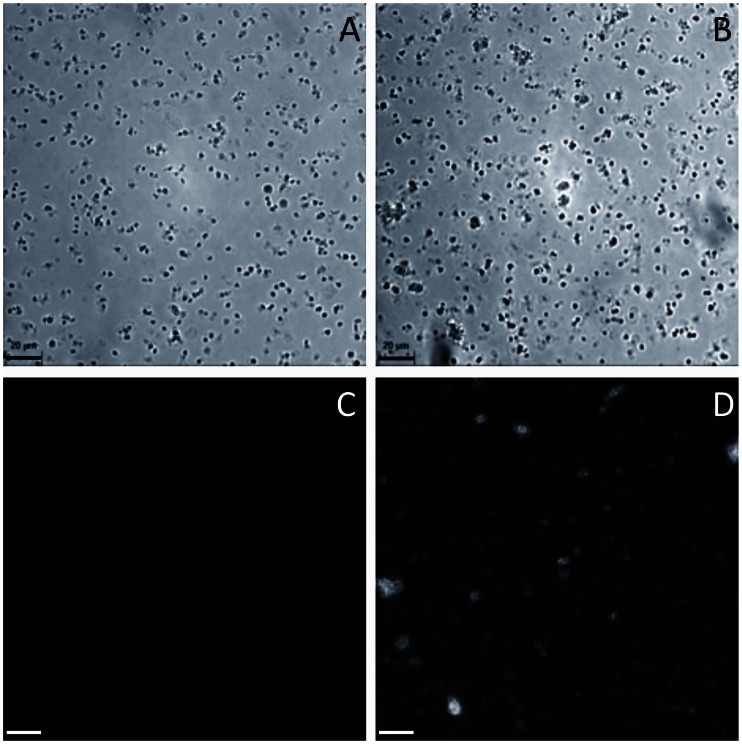
Real-time immunofluorescence study of P-selectin surface expression on platelets adhered to VWF and stimulated by thrombin. Human blood platelets were perfused on human VWF at 1800 s^−1^ for 20 minutes and activated with thrombin as described in Materials and Methods. Then an Alexa fluor 488 conjugated antibody to P-selectin was perfused in the flow channels. Phase-contrast images are shown for non-stimulated platelets (Panel A) and stimulated platelets (Panel B). Following immunofluorescent labeling, thrombin-activated platelets displayed positive P-selectin staining (Panel D), whereas unactivated platelets remained negative (Panel C). Images were recorded using a Qimaging Rolera camera. Bar = 20 µm.

Direct immunofluorescence staining in situ in the flow channel was used to study the activation status of platelets translocating on VWF. P-selectin staining was absent without thrombin activation, but it was observed following thrombin activation (Figure 5). This result was confirmed by flow cytometry analysis of P-selectin and Alexa 488 fibrinogen binding performed on suspensions of platelets collected at the exit of the flow chamber exposure. Results indicated that exposure to flow maintained the platelet αIIbβ3 integrin in a non-activated state, and that it was fully activable following activation by thrombin (Figure S1).

### Proplatelet Formation in a Model of Chemotherapy-induced Thrombocytopenia

**Figure 6:**
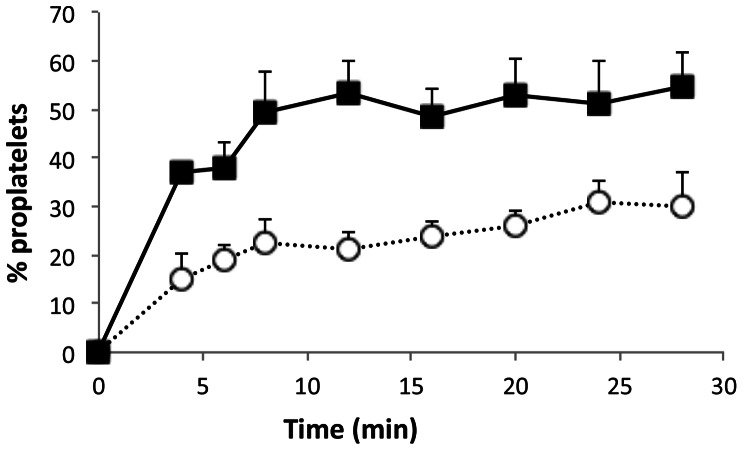
5 FU-induced thrombocytopenia model of proplatelet formation. A thrombocytopenic mouse model was developed following a single injection of 5-FU in mice. Shear exposure to VWF was performed 7 days after 5-FU administration, using platelets isolated from blood samples as described in the legend to Figure 3. The data showing determination of proplatelets are shown as means ± SEM of the % of proplatelets in 5-FU thrombocytopenic mice (closed squares) *vs* untreated mice (open circles) (n = 4 experiments).

We then examined whether proplatelets were also visible in a situation of mild thrombocytopenia. To this end, *Vwf^ +/+^* mice received a single 5-FU injection, resulting in approximately 50% reduction in platelet counts and mean platelet volumes (Figure S2). Mouse blood was sampled 7 days after 5-FU injection representing the end of the thrombocytopenic phase, and the proportion of proplatelets was determined in conditions of exposure to shear and VWF (Figure 6). A significantly higher percentage of proplatelets was obtained from 5-FU treated thrombocytopenic *Vwf^ +/+^* mice than from untreated mice (6 to 20 min-time points, p<0.05). In addition, when *Vwf ^−/−^* mice were treated with 5-FU, 20–25% more proplatelets were observed in 5-FU treated *Vwf ^−/−^* mice than in untreated *Vwf ^−/−^* mice (data not shown). These results suggest that *Vwf ^−/−^* and *Vwf^ +/+^* mice recovering from thrombocytopenia produced young proplatelets and young platelets. However, in 5-FU treated *Vwf ^−/−^* mice, the % of proplatelets remained much higher (92% at 20 min) than in 5-FU treated *Vwf^ +/+^* mice (53%). These additive effects may indicate that the role of VWF and the increased production of young proplatelets 7 days after chemotherapy may be complementary during terminal platelet production.

### Platelets from a Patient with Type 3 von Willebrand Disease Display Cytoskeleton Modifications

**Figure 7:**
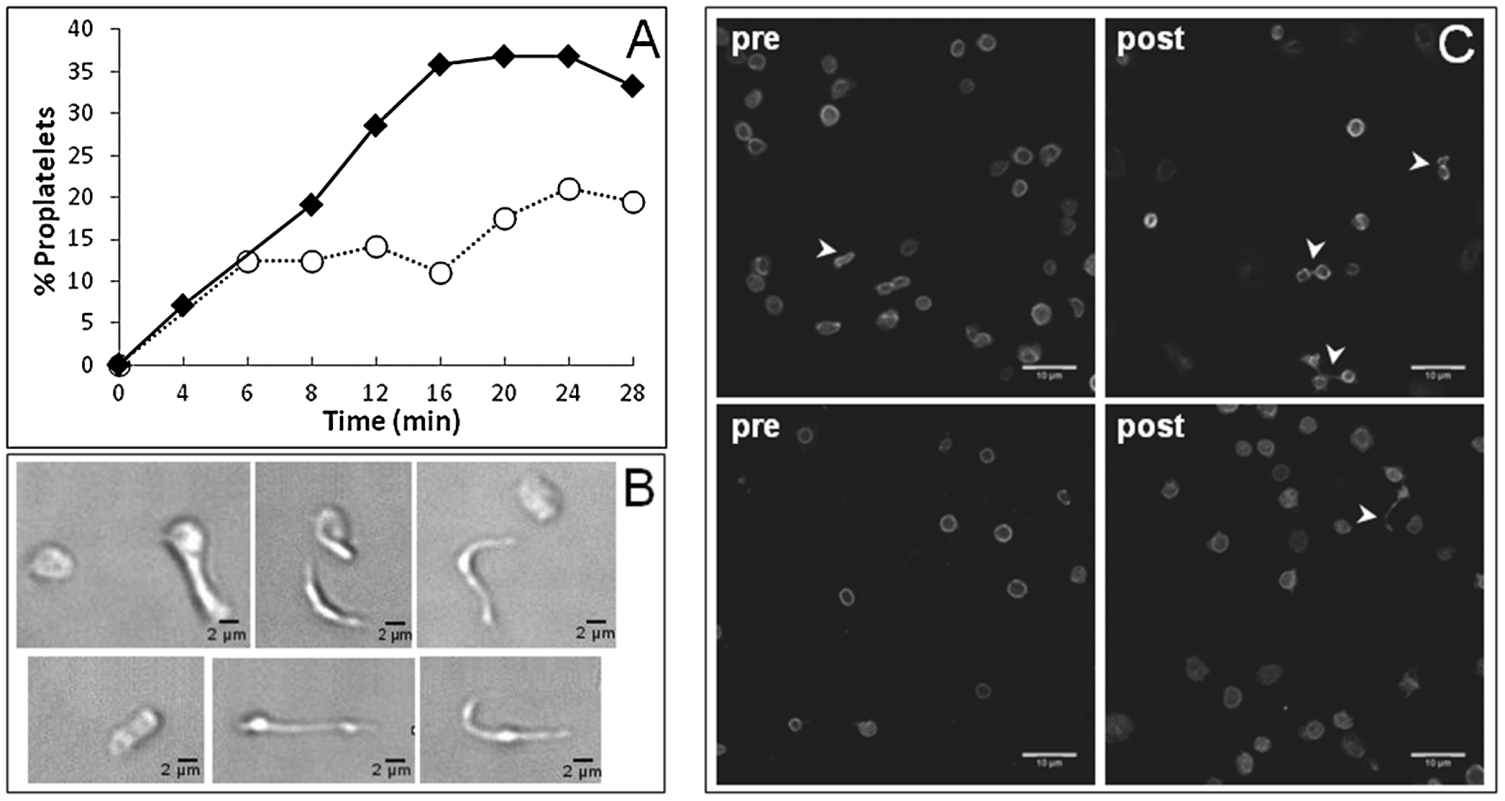
Characterization of proplatelets in human blood from a healthy control and a patient with type 3 von Willebrand disease. Human washed platelets were perfused on human VWF at 1800 s^−1^ and images were recorded continuously. Panel A compares the % of proplatelets (see Figure 3 legend) obtained from washed platelets isolated from a patient with type 3 von Willebrand disease (closed diamonds) and a healthy control (open circles). In this experiment, 180 G/L platelets were perfused on VWF. Panel B displays different morphological states of reversibly adherent control proplatelets, including from left to right: discoid platelets, dumbbell-shaped, “figure 8”, extended and multibodied proplatelets (bar = 2 µm). Panel C depicts tubulin organization in platelets from the patient with type 3 von Willebrand disease (upper panels) and a control donor (lower panels) in two situations: immediately prior to shear exposure and in effluents after shear exposure. Confocal microscopy was used to identify proplatelets (arrowheads) and discoid platelets. Bar = 10 µm.

Type 3 von Willebrand disease is a very rare inherited disorder characterized by a total lack of VWF synthesis and, thus, the absence of VWF from the vascular compartment (endothelial cells and subendothelium) as well as from MKs and platelets. Washed platelets from such a patient were exposed *ex vivo* to a VWF-coated surface at a shear rate of 1800 s^−1^. Control donor post-shear samples contained more proplatelets than pre-shear samples which contained discoid platelets. Relative to healthy control platelets (Figure 7A, 7B and Video S3), the patient’s blood contained more proplatelets. As shown in Figure 7C, there was a difference between pre- and post-shear type 3 VWD patient blood samples. Pre-shear (i.e. in static conditions) patient’s sample contained some proplatelets resembling figure 8 or dumbbells. The proplatelets were not only more abundant in the patient’s post-shear sample, but they were also in a state of more advanced fragmentation.

## Discussion

Platelets are small anuclear cells produced by terminal differentiation of mature MKs originating from bone marrow precursors. To examine the possible role of VWF in this terminal phase of thrombopoiesis, we used a *Vwf ^−/−^* mouse model characterized by a total absence of VWF. Platelets isolated from *Vwf ^−/−^* mouse blood were compared to those isolated from *Vwf^ +/+^* mouse blood in a real-time flow assay designed to examine the transition from proplatelet to platelet formation *ex vivo*, in the presence of VWF and high shear rates similar to those used in a previous report showing proplatelet and platelet formation from mature MKs [13]. Others have recently suggested that large round fragments (preplatelets) and proplatelets are transient states, and are irreversibly converted into discoid platelets through an unknown mechanism [14], [16]. Based on our previous observations of such intermediates during MK fragmentation at high shear rates on VWF, we reasoned that the processing of platelets during a long static incubation of blood, reported by Schwertz & al [16] could be favored by exposure to high shear rates on VWF. Furthermore, this study provided an opportunity to elucidate the role of VWF present on vessel walls during thrombopoiesis.

Evidence for a role of VWF in terminal platelet production in high-shear conditions was obtained during *ex vivo* perfusion, and on some occasions required *in vivo* pre-treatment of mice. The main results of this study are the following: 1) *Ex vivo* exposure of *Vwf ^−/−^* mouse platelets to high shear rates on a VWF surface resulted in a significantly higher percentage of proplatelets than when *Vwf^+/+^* mouse platelets were used, suggesting that platelet exposure to VWF present in the vasculature may be essential for normal thrombopoiesis. 2) This increased proportion of proplatelets was partially corrected by hydrodynamic injection of VWF cDNA to *Vwf ^−/−^* mice, as it was intermediate between *Vwf^ +/+^* mice and untreated *Vwf ^−/−^* mice. 3) The fraction of regenerating platelets *in vivo* following chemotherapy-induced thrombocytopenia was enriched in proplatelets compared to untreated mice, similar to what was observed in untreated *Vwf ^−/−^* mice. 4) The blood of a patient with type 3 von Willebrand disease contained more proplatelets than that of a healthy control.

Interestingly, we found that VWF-deficient mouse blood exposed to a high shear rate contained a higher proportion of proplatelets than did *Vwf^ +/+^* blood. Two mechanisms might explain this effect. Firstly, comparison of pre-shear and post-shear samples indicated that shear exposure on VWF allows concentrating rare events, such as the presence of proplatelets in circulating blood. Proplatelets were detected after passage in the microfluidic system, whereas they were less visible in the pre-shear samples. The different proportion of proplatelets between pre-shear and post-shear samples was more important in *Vwf ^−/−^* mouse and in type 3 VWD patient blood than in *Vwf^ +/+^* mice and in healthy control blood. Shear exposure allowed proplatelets to adopt different shapes (a dumbbell shape or a more complex structure with several cell bodies), and also the breakage of proplatelets into platelets, reminiscent of what was seen during the last steps of MK fragmentation into platelets. Secondly, e*x vivo*, *Vwf ^−/−^* blood platelets exposed to a high shear rate behave as “naive” platelets encountering VWF for the first time [22]. We propose that *in vivo* proplatelet breakage in platelets may be slower in *Vwf ^−/−^* mice, due to the absence of vascular VWF. This step would be accelerated *ex vivo* by passage at 1800 s^−1^ on a VWF-coated surface.

This process occurred without platelet activation, as shown by continuous platelet translocation on the surface. The low level of platelet activation observed in these experiments allowed us to rule out an effect of VWF release from alpha-granules in *Vwf^ +/+^* platelets. In separate experiments, actin labeling confirmed that platelets were not activated but displayed stress fibers following the addition of thrombin (data not shown), indicating that both proplatelets and discoid platelets were functional. Thus, the increased percentage of proplatelets generated *ex vivo* from *Vwf* ^−/−^ blood compared to *Vwf ^+/+^* blood appeared to be related to *in vivo* contact of proplatelets with VWF on the vessel wall. To confirm this, we performed hydrodynamic injections of *Vwf* cDNA in *Vwf ^−/−^* mice. We postulated that although this procedure is not considered to replenish VWF stores in cellular/vascular compartments, the 3- to 4-fold increase in VWF plasma concentrations could result in some VWF deposition on vessel walls and thereby allow more complete terminal platelet production. Indeed, the proportion of proplatelets obtained from *Vwf ^−/−^* mice was significantly lower after hydrodynamic injection with *Vwf* cDNA than with the empty vector.

The present work extends our previous findings on the dynamic process of platelet formation from MKs at high shear rates [13]. Recent data indicate that VWF is present on stimulated [23], [24] and even healthy endothelium, whether released from storage granules or by exocytosis [25]. Our present findings show that high shear rates are essential for thrombopoiesis, and that VWF present on the vessel wall is required for terminal platelet production. Our study suggests that VWF may also allow a continuous transition from proplatelets to discoid platelets. These proplatelets may act as a reservoir of platelets ready to replace discoid platelets that have been activated at a site of vascular injury. This model is compatible with the relatively small number of mature MKs present in human bone marrow (10/mm^2^) compared to the large number (150–400 G/L) of circulating platelets. Thus, we propose a new role of VWF in regulating the final stage of platelet production via its action in the circulation at high shear rates. This is in addition to its main function in initiating platelet adhesion and bleeding arrest. Our findings may have direct pathophysiological implications for macrothrombocytopenia in patients with congenital GPIb-V-IX deficiency [26], in whom the absence of GPIb (the primary VWF receptor) prevents complete processing of proplatelets to functional discoid platelets *in vivo,* despite a normal VWF content in vessel walls. Recently, a role of the GPIb-V-IX complex in proplatelet formation was identified in mouse models [27]. However, it may appear surprising that in type 3 von Willebrand disease patients (and in *Vwf ^−/−^* mouse blood) with complete VWF deficiency, platelet counts and platelet size appear to be normal. This does not, however, exclude a role of VWF *in vivo*. As recently supported by mathematical modeling, proplatelets may exist in blood at a low concentration, and their fragmentation into platelets may be very rapid [28]. Thus, the platelet count and size would not necessarily reflect delayed conversion of proplatelets into platelets.

Our results obtained after 5-FU chemotherapy suggest that VWF may play an unsuspected role in the regulation of platelet production. 5-FU selectively kills cycling progenitor cells, including megakaryocyte progenitors [29]. After an initial drop in the platelet count following 5-FU treatment (nadir reached on day 7), the platelet count re-increases 11 to 19 days after treatment [30]. It has been reported that, 11 days after 5-FU treatment, young platelets in mouse blood display larger microtubule coils than untreated platelets [31]. An increased population of mature MKs appears after 7 days in 5-FU-treated mice, compared to 14 days in untreated mice [32]. We suspected that circulating proplatelets generated *in vivo* from an increased population of mature MKs would be detectable 7 days after 5-FU treatment. Indeed, we found an increased proportion of proplatelets in 5-FU-treated thrombocytopenic *Vwf^ +/+^* mice, as measured *ex vivo* upon exposure to shear stress and on VWF. Interestingly, in *Vwf ^−/−^* mice, 5-FU-induced thrombocytopenia also resulted in a marked increase of terminal platelet production as shown by >90% proplatelets compared to 53% in 5-FU treated *Vwf^ +/+^* mice. This strongly suggests that terminal platelet production is regulated by VWF, an effect even observed during the increased production of young platelets 7 days after chemotherapy.

Besides complete VWF deficiency, variable levels of macrothrombocytopenia identified in type 2B von Willebrand disease, a disorder characterized by gain-of-function mutations [33], [34], point to a role of VWF in the regulation of platelet production. We have previously observed macrothrombocytopenia in a mouse model of type 2B von Willebrand disease [35]. In addition, proplatelet formation from MK exposed to a high shear rate on type 2B mutant recombinant VWF was impaired compared to wild-type recombinant VWF [13]. Finally, in case of VWF deficiency, other receptor-ligand interactions that are known to occur at low shear rates might compensate for the absence of GPIb interaction with VWF at a high shear rate. In particular, fibronectin may also act as a GPIb ligand and take over the fragmentation of proplatelets into platelets in low shear rate conditions.

## Supporting Information

Figure S1 **Thrombin activation of effluents of mouse platelet samples prior and after shear exposure.** Platelet suspensions from *Vwf^ +/+^* mouse blood were collected before exposure to shear (left panels) and after shear exposure (right panels) for analysis by flow cytometry before (thin lines) and after activation by thrombin (bold lines). Upper panel: binding of Alexa 488 fibrinogen, lower panel: immunolabelling with anti-P-selectin (CD62P). The non immune control is shown as a shaded area. Note that pre-shear samples overlapped with post-shear samples in each condition. Platelets collected at the channel exit formed a heterogeneous mixture of those sheared on the VWF surface and those that were not sheared. It is shown that an increase in fibrinogen and CD62P binding upon platelet stimulation with exogenous thrombin was observed following shear exposure and similar to that observed with platelets kept in static conditions.
(DOCX)

Figure S2 **A thrombocytopenic mouse model was developed following a single injection of 5-FU in 6 mice (closed squares) *vs* 5 control mice injected with saline (open circles).** Platelet counts (panel A) and median platelet volumes (panel B) were assessed daily, and indicated that 5-FU induced a mild thrombocytopenia that reached its nadir at day 7, and was reversed at day 10 (mean±SEM).
(DOCX)

Video S1 **Adhesion of Vwf ^−/−^ platelets. Mouse platelets were perfused at a shear rate of 1800 s^−1^ for 35 min on VWF-coated coverslips and adherent platelets were visualized on the VWF surface.** Digital images were recorded at 0.25 image/s using “Replay” and “Histolab” software. For visualization, “Archimed” software (Microvision Instruments) was used to grab frames and record at a velocity of 10 images/s (40-fold acceleration). The set-up allows analyzing the changes of platelet morphology during their crossing of one field (an event that may extend up to 10 min). The release of a small discoid platelet from a proplatelet can be visualized in real-time: in the lower third of the image on the left, entering the field at 16′54″ is a dumbbell-shaped proplatelet (arrowhead) with a thin extension at its rear; the proplatelet translocates along the field and its extension corresponds to a small platelet (arrow at 18′06″), which is released at 20′42″ (arrow) from the original proplatelet (arrowhead). Bar is 20 µm.
(AVI)

Video S2 **Adhesion of Vwf^ +/+^ platelets. Proplatelets and large condensed platelets are present.** Notice in particular the dumbbell-shaped proplatelet (arrowhead) that translocates on the surface to adopt a more condensed shape, then extends again as a multibodied proplatelet, followed by the appearance of a small discoid extension at 24′05″ (arrow) that continues to extend progressively and may correspond to a small discoid platelet (arrow) about to be released by a larger proplatelet (arrowhead) on the last frames. Note that reversibly adherent *Vwf ^−/−^* proplatelets are larger than *Vwf^ +/+^* proplatelets. Bar is 20 µm.
(AVI)

Video S3 **Adhesion of human platelets. Human platelets from a healthy volunteer were perfused at a shear rate of 1800 s^−1^ for 18 min on VWF-coated coverslips and platelets were visualized in real-time on the VWF surface.** Different morphologies of proplatelets are present. For example, in the upper part of the field, during the first 18 seconds of the video, notice an elongated newly formed platelet that translocates along the field and folds like a knot, then unfolds to appear as an extended platelet. In the upper third of the field, entering at time 37″ until 57″, notice how another newly formed platelet displays different shapes during its translocation on the surface. Digital images were recorded at 0.25 image/s using Replay software (Microvision Instruments). For visualization of the movie, frames were grabbed so as to accelerate to a velocity of 10 images/s. Bar represents 20 µm.
(AVI)
