# Supplementary material for: Terminal Platelet Production is Regulated by Von Willebrand Factor
Source: PLoS One. 2013 May 30;8(5):e63810. doi: 10.1371/journal.pone.0063810 (PMC3667798; doi:10.1371/journal.pone.0063810)
Supplement: Figure S1 — Thrombin activation of effluents of mouse platelet samples prior and after shear exposure. Platelet suspensions from Vwf +/+ mouse blood were collected before exposure to shear (left panels) and after shear exposure (right panels) for analysis by flow cytometry before (thin lines) and after activation by thrombin (bold lines). Upper panel: binding of Alexa 488 fibrinogen, lower panel: immunolabelling with anti-P-selectin (CD62P). The non immune control is shown as a shaded area. Note that pre-shear samples overlapped with post-shear samples in each condition. Platelets collected at the channel exit formed a heterogeneous mixture of those sheared on the VWF surface and those that were not sheared. It is shown that an increase in fibrinogen and CD62P binding upon platelet stimulation with exogenous thrombin was observed following shear exposure and similar to that observed with platelets kept in static conditions. (DOCX) [file pone.0063810.s001.docx]

**Supplementary Figure S1**

*Thrombin activation of effluents of mouse platelet samples prior and after shear exposure*

**

**
