# Supplementary material for: Terminal Platelet Production is Regulated by Von Willebrand Factor
Source: PLoS One. 2013 May 30;8(5):e63810. doi: 10.1371/journal.pone.0063810 (PMC3667798; doi:10.1371/journal.pone.0063810)
Supplement: Figure S2 — A thrombocytopenic mouse model was developed following a single injection of 5-FU in 6 mice (closed squares) vs 5 control mice injected with saline (open circles). Platelet counts (panel A) and median platelet volumes (panel B) were assessed daily, and indicated that 5-FU induced a mild thrombocytopenia that reached its nadir at day 7, and was reversed at day 10 (mean±SEM). (DOCX) [file pone.0063810.s002.docx]

**Supplementary Figure S2**

*Platelet counts and median platelet volumes in a thrombocytopenic mouse model*

**
